# Supplementary material for: Utilizing wearable sensors for continuous and highly-sensitive monitoring of reactions to the BNT162b2 mRNA COVID-19 vaccine
Source: Commun Med (Lond). 2022 Mar 14;2:27. doi: 10.1038/s43856-022-00090-y (PMC9053261; doi:10.1038/s43856-022-00090-y)
Supplement: Supplementary file 1 — Supplementary Information [file 43856_2022_90_MOESM1_ESM.pdf]

Supplementary Materials for:

Utilizing wearable sensors for continuous and highly-sensitive monitoring of reactions to the BNT162b2 mRNA COVID-19 vaccine

Yftach Gepner, PhD[1,+], Merav Mofaz, MSc[2], Shay Oved, MSc[2], Matan Yechezkel, MSc[2], Keren Constantini, PhD[1], Nir Goldstein, PhD[1], Arik Eisenkraft, MD, PhD[3,4,], Erez Shmueli, PhD[2,5,+], and Dan Yamin, PhD[2,6,+,*]

[1]Department of Epidemiology and Preventive Medicine, School of Public Health, Sackler Faculty of Medicine, and Sylvan Adams Sports Institute, Tel-Aviv University, Tel-Aviv, Israel

[2]Department of Industrial Engineering, Tel-Aviv University, Tel-Aviv, Israel

[3]The Institute for Research in Military Medicine, the Hebrew University Faculty of Medicine, Jerusalem, Israel

[4]Chief Medical Officer, Biobeat Technologies Ltd., Petah-Tikva, Israel

[5]MIT Media Lab, Cambridge MA, USA

[6]Center for Combatting Pandemics, Tel-Aviv University, Tel-Aviv, Israel

[+] These authors contributed equally

* Corresponding author. Email: dan.yamin@gmail.com

Table of Contents

Supplementary Figures 2

Supplementary Figure S1 2

Supplementary Figure S2 4

Supplementary Figure S3 5

Supplementary Figure S4. 6

Supplementary Figure S5. 8

Supplementary Figure S6. 9

Supplementary Tables 10

Table S1 10

Supplementary Figures

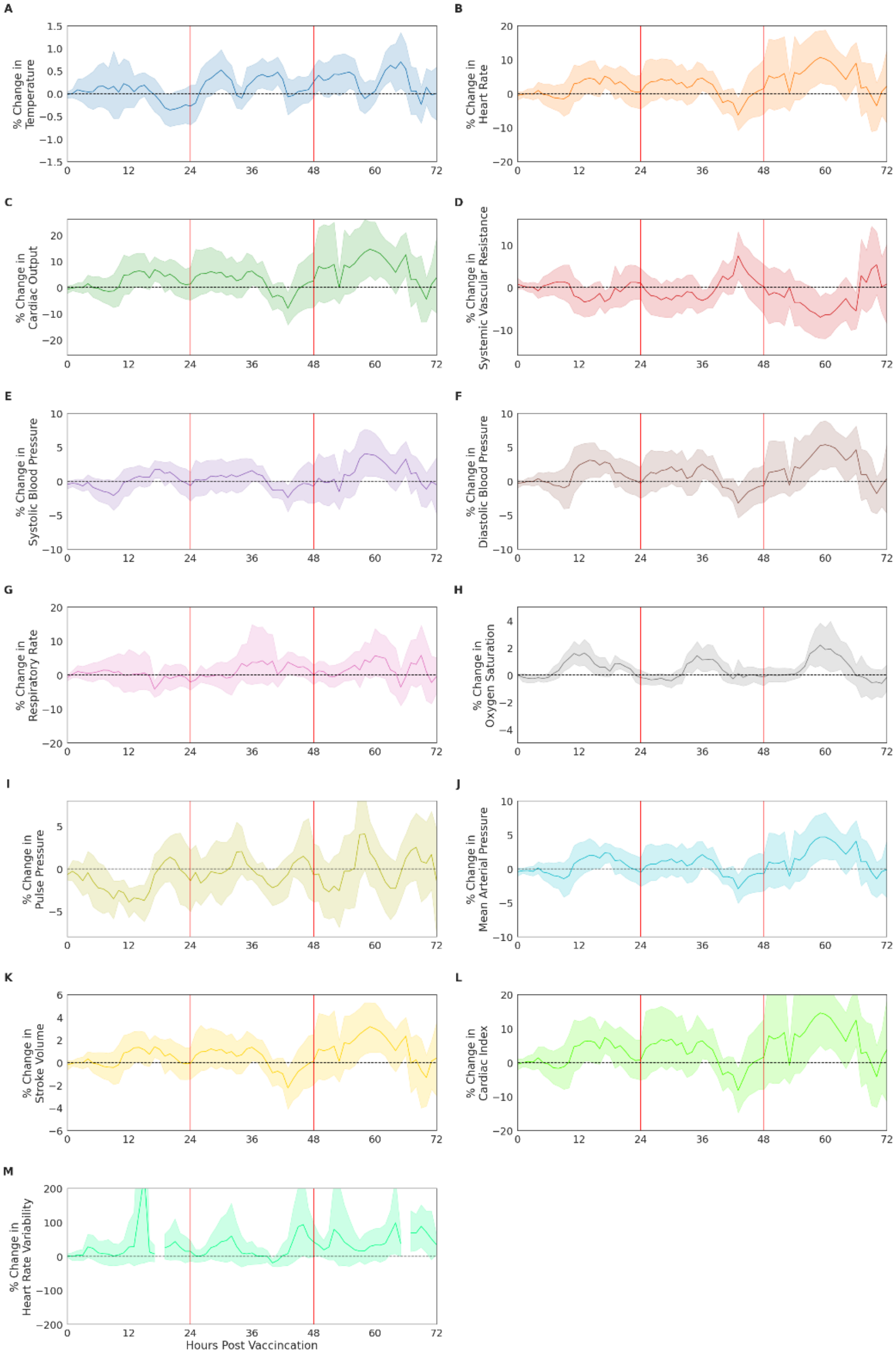

Supplementary Figure S1. Percentage of change in respiratory, cardiovascular, and physiological indicators recorded by the chest-patch sensor after receiving the first vaccine dose, compared to their levels observed on the day prior vaccination: (**A**) skin temperature, (**B**) heart rate, (**C**) cardiac output, (**D**) systemic vascular resistance, (**E**) systolic blood pressure, (**F**) diastolic blood pressure, (**G**) respiratory rate, (**H**) oxygen

saturation, (**I**) pulse arterial pressure, (**J**) mean arterial pressure, (**K**) stroke volume, (**L**) cardiac index, and (**M**) heart rate variability. Mean values are depicted as solid lines, 90% confidence intervals are presented as shaded regions, and horizontal dashed lines represent no change compared to the levels observed on the day prior vaccination.

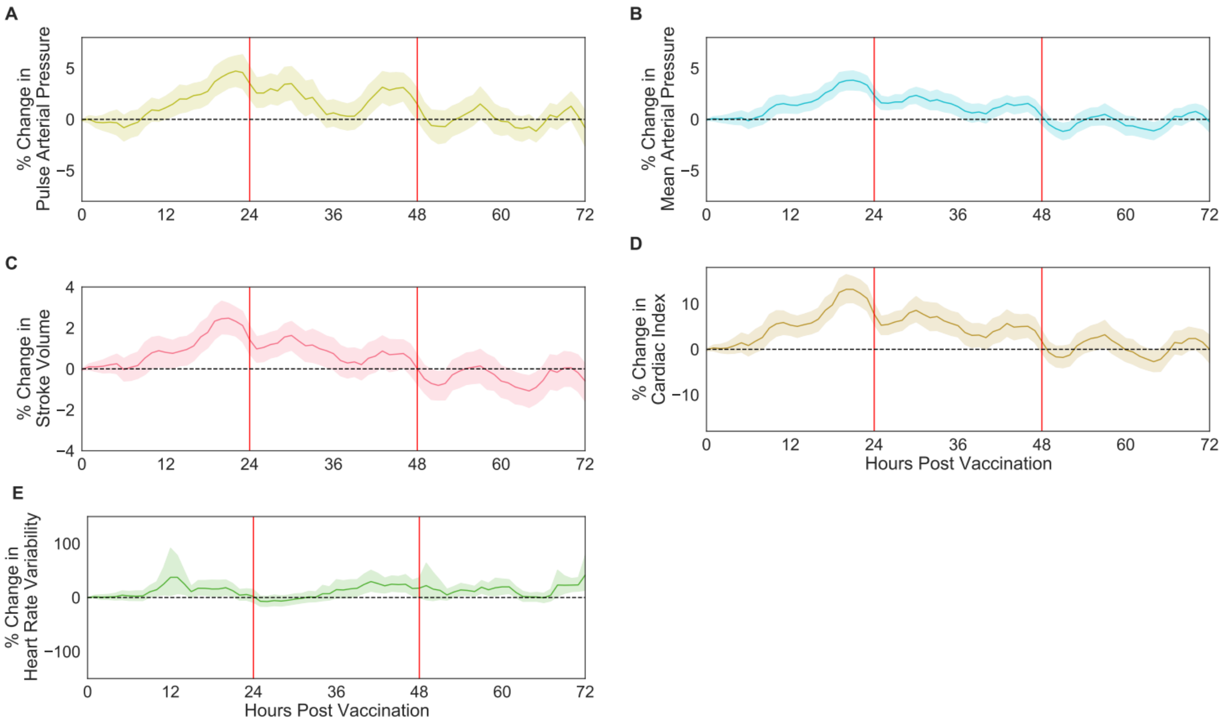

Supplementary Figure S2. Percentage of change in respiratory, cardiovascular, and physiological chest-patch indicators recorded by the chest-patch sensor after receiving the second vaccine dose, compared to their levels observed on day prior vaccination: (**A**) pulse arterial pressure, (**B**) mean arterial pressure, (**C**) stroke volume, (**D**) cardiac index, (**E**) heart rate variability. Mean values are depicted as solid lines, 90% confidence intervals are presented as shaded regions, and horizontal dashed lines represent no change compared to the levels observed on day prior vaccination.

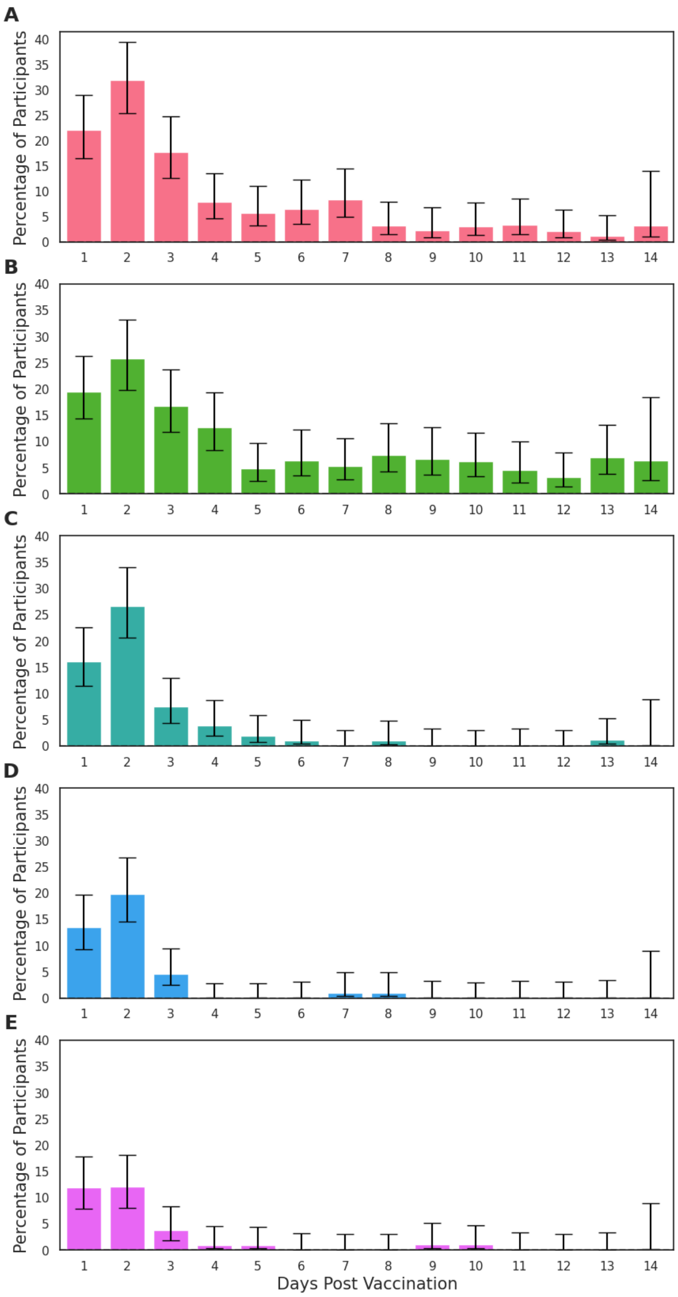

Supplementary Figure S3. Most frequent local and systemic reactions reported by participants through the mobile application after receiving the second vaccine dose. (**A**) fatigue, (**B**) headache, (**C**) muscle pain, (**D**) fever, and (**E**) chills. Error bars represent 90% confidence intervals. Horizontal dashed lines represent no change compared to the levels observed on day prior vaccination.

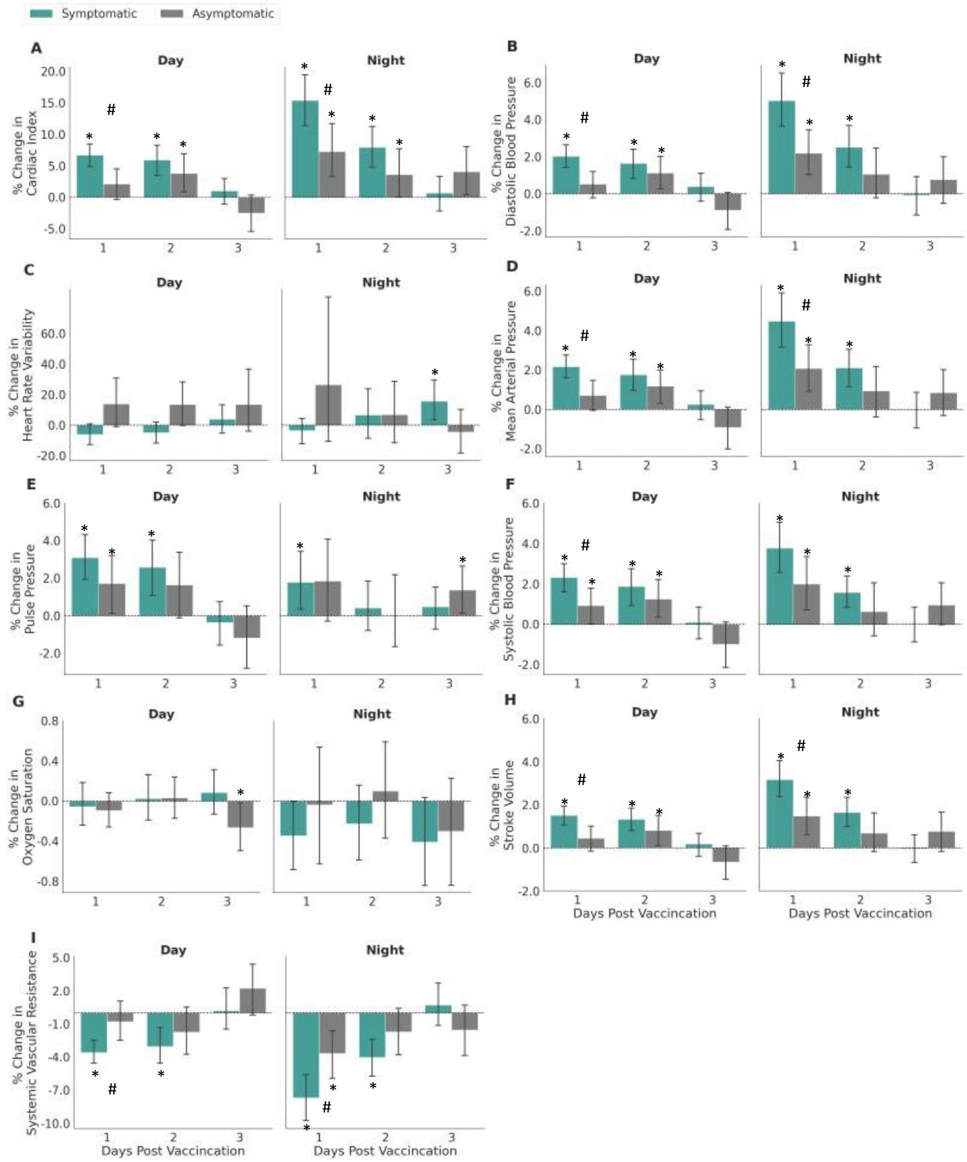

Supplementary Figure S4. Percentage of change in chest-patch indicators for participants who reported at least one local or systemic reaction during the two days post second vaccination (symptomatic) and those who did not reported any reaction (asymptomatic), during days and nights: (**A**) cardiac index, (**B**) diastolic blood pressure, (**C**) heart rate variability, (**D**) mean arterial pressure, (**E**) pulse arterial pressure, (**F**) systolic blood pressure, (**G**) oxygen saturation, (**H**) stroke volume, and (**I**) systemic vascular resistance. Error bars represent 90% confidence intervals. Horizontal dashed lines represent no change compared to the levels observed on the day prior vaccination. Significant differences compared to the levels observed on the day

prior vaccination at a 0.05 level are marked with ∗. Significant differences between symptomatic and asymptomatic participants at a 0.05 level are marked with #.

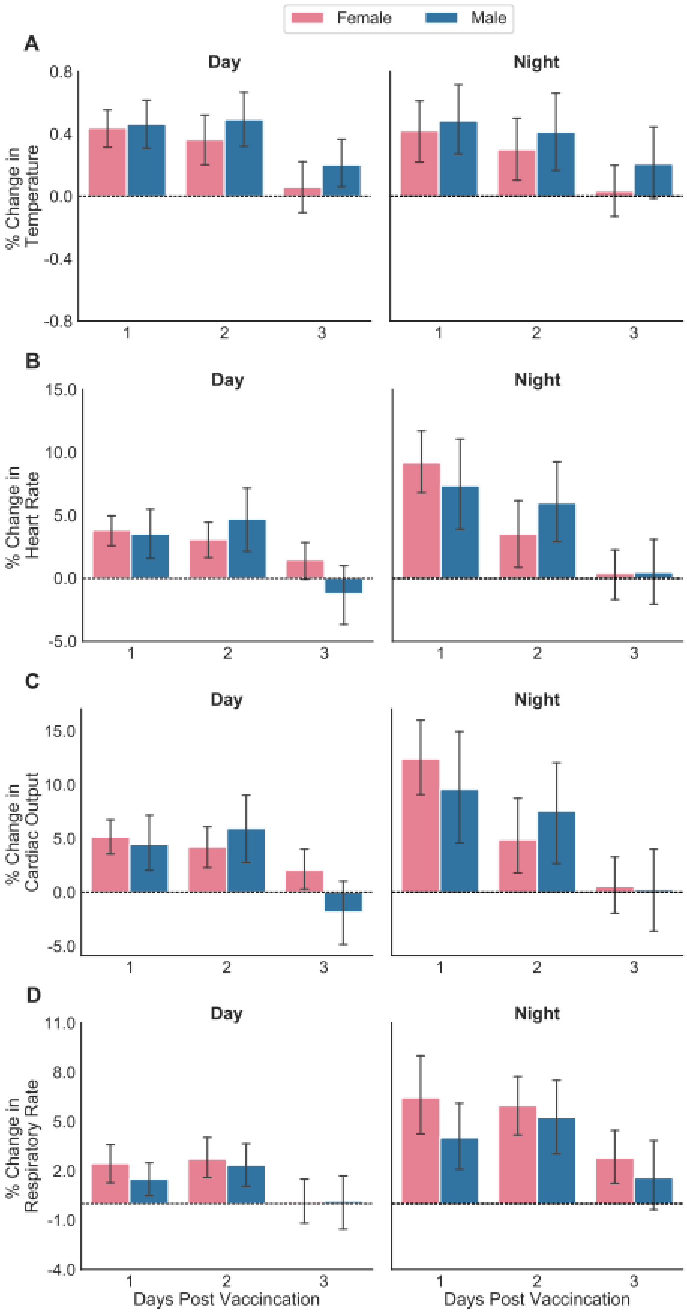

Supplementary Figure S5. Percentage of change in chest-patch indicators after receiving the second vaccine dose, for participants divided by gender during the daytime and the nighttime: (**A**) skin temperature, (**B**) heart rate, (**C**) cardiac output, and (**D**) respiratory rate. Error bars represent 90% confidence intervals. Horizontal dashed lines represent no change compared to the levels observed on the day prior vaccination.

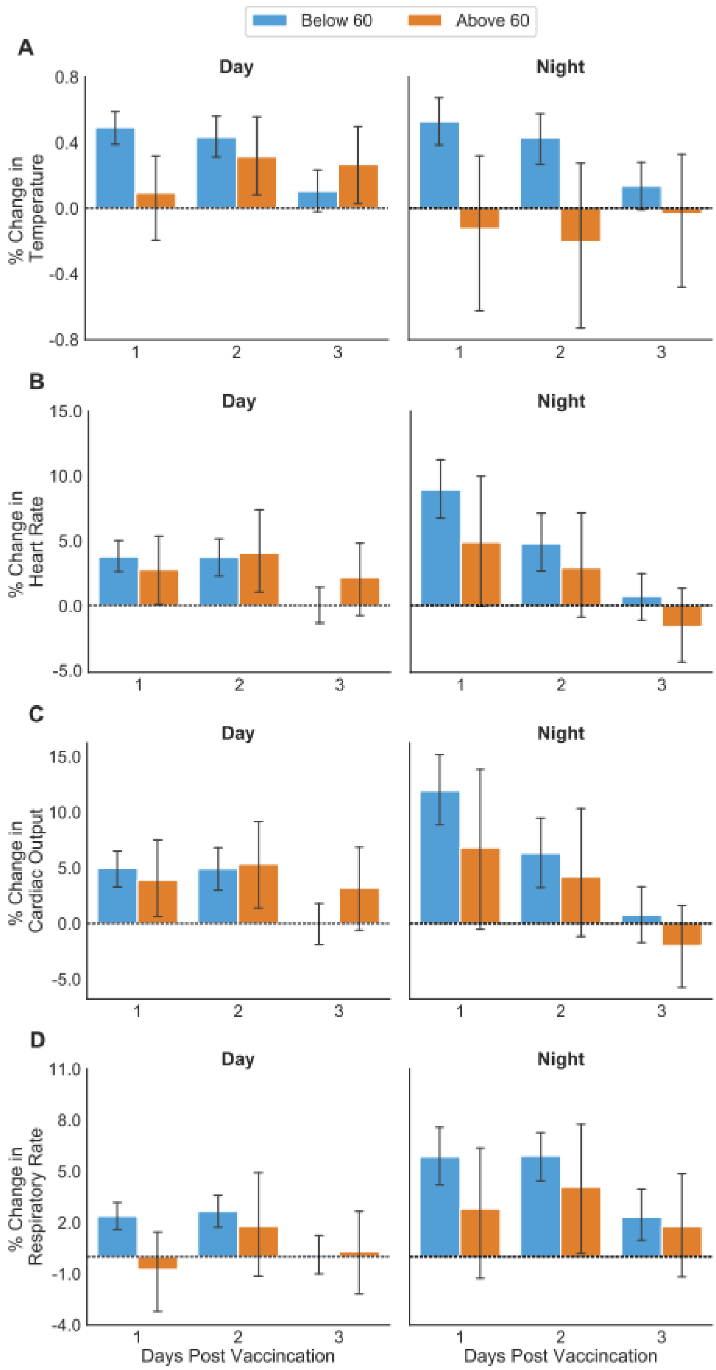

Supplementary Figure S6. Percentage of change in chest-patch indicators after receiving the second vaccine dose, for participants divided by age group during the daytime and the nighttime: (**A**) skin temperature, (**B**) heart rate, (**C**) cardiac output, and (**D**) respiratory rate. Error bars represent 90% confidence intervals. Horizontal dashed lines represent no change compared to the levels observed on the day prior vaccination.

Supplementary Tables

Table S1. Descriptive statistics for chest-patch indicators for second vaccine dose. Each row represents a single chest-patch indicator. The two left columns represent the mean value for each indicator ± one standard error during the day prior vaccination. The columns on the right represent the change in percentage for each indicator ± one standard error, for each of the three days post vaccination during nighttime and during daytime.

	**Day prior vaccination**		**% Change - Day 1**		**% Change - Day 2**		**% Change - Day 3**	
	**Day**	**Night**	**Day**	**Night**	**Day**	**Night**	**Day**	**Night**
**Skin Temperature (c)**	37±0.03	37±0.03	0.45±0.06	0.42±0.07	0.12±0.07	0.45±0.09	0.35±0.1	0.11±0.08
**Heart Rate (bpm)**	80±0.75	67±0.70	3.69±0.65	3.79±0.81	0.26±0.78	8.44±1.29	4.54±1.24	0.42±1.00
**Cardiac Output (L/min)**	6.45±0.08	5.13±0.07	4.84±0.89	4.97±1.09	0.32±1.04	11±1.80	6.01±1.74	0.4±1.37
**Systemic Vascular Resistance** $(\mathbf{dynes \cdot sec \cdot cm^{-5}})$	1255±15	1453±21	-2.45±0.59	-2.36±0.71	0.66±0.82	-5.26±1.03	-2.46±0.97	0.93±0.98
**Systolic Blood Pressure (mmHg)**	128±1.22	119±1.20	1.7±0.32	1.56±0.39	-0.08±0.39	2.79±0.53	1.13±0.47	0.0±0.40
**Diastolic Blood Pressure (mmHg)**	82±0.76	75±0.85	1.48±0.29	1.35±0.34	-0.0±0.36	3.64±0.62	1.78±0.58	-0.26±0.48
**Respiratory Rate (br/min)**	16±0.08	16±0.15	2.04±0.48	2.56±0.54	0.13±0.63	5.46±0.94	5.67±0.86	2.25±0.80
**Oxygen Saturation (%)**	97±0.08	96±0.13	-0.12±0.08	-0.0±0.09	-0.04±0.09	-0.16±0.18	-0.18±0.18	-0.28±0.20
**Pulse Pressure (mmHg)**	46±0.82	44±0.82	2.24±0.51	2.19±0.64	-0.11±0.57	1.54±0.69	0.39±0.64	0.68±0.47
**Mean Arterial Pressure (mmHg)**	97±0.85	90±0.90	1.58±0.29	1.46±0.35	-0.03±0.36	3.25±0.57	1.52±0.52	-0.13±0.44
**Stroke Volume (mL/beat)**	80±0.80	76±0.87	0.95±0.27	0.9±0.33	-0.22±0.36	2.07±0.47	0.92±0.46	-0.32±0.45
**Cardiac Index (L/min/m2)**	3.57±0.06	2.85±0.05	4.83±0.89	4.85±1.11	0.33±1.06	11±1.81	5.89±1.73	0.54±1.38
**Heart Rate Variability (%)**	6.89±0.26	4.78±0.23	0.01±4.07	0.13±4.00	7.71±5.41	13±10	14±9.31	7.95±6.15
