# Supplementary material for: Utilizing wearable sensors for continuous and highly-sensitive monitoring of reactions to the BNT162b2 mRNA COVID-19 vaccine
Source: Commun Med (Lond). 2022 Mar 14;2:27. doi: 10.1038/s43856-022-00090-y (PMC9053261; doi:10.1038/s43856-022-00090-y)
Supplement: Supplementary file 3 — Reporting Summary [file 43856_2022_90_MOESM3_ESM.pdf]

nature research

Corresponding author(s): Dr. Dan Yamin
Last updated by author(s): Feb 1, 2022

Reporting Summary

Nature Research wishes to improve the reproducibility of the work that we publish. This form provides structure for consistency and transparency in reporting. For further information on Nature Research policies, see our Editorial Policies and the Editorial Policy Checklist.

Statistics

For all statistical analyses, confirm that the following items are present in the figure legend, table legend, main text, or Methods section.

n/a	Confirmed	
☐	☒	The exact sample size (n) for each experimental group/condition, given as a discrete number and unit of measurement
☐	☒	A statement on whether measurements were taken from distinct samples or whether the same sample was measured repeatedly
☐	☒	The statistical test(s) used AND whether they are one- or two-sided<br>*Only common tests should be described solely by name; describe more complex techniques in the Methods section.*
☐	☒	A description of all covariates tested
☐	☒	A description of any assumptions or corrections, such as tests of normality and adjustment for multiple comparisons
☐	☒	A full description of the statistical parameters including central tendency (e.g. means) or other basic estimates (e.g. regression coefficient) AND variation (e.g. standard deviation) or associated estimates of uncertainty (e.g. confidence intervals)
☐	☒	For null hypothesis testing, the test statistic (e.g. F, t, r) with confidence intervals, effect sizes, degrees of freedom and P value noted<br>*Give P values as exact values whenever suitable.*
☐	☒	For Bayesian analysis, information on the choice of priors and Markov chain Monte Carlo settings
☐	☒	For hierarchical and complex designs, identification of the appropriate level for tests and full reporting of outcomes
☐	☒	Estimates of effect sizes (e.g. Cohen's d, Pearson's r), indicating how they were calculated

Our web collection on statistics for biologists contains articles on many of the points above.

Software and code

Policy information about availability of computer code

Data collection	Data were collected using custom code, programmed using Python 3.7 environment. The storage server runs a CentOS operating system.
Data analysis	Data were analyzed using Python 3.7 programming environment.

For manuscripts utilizing custom algorithms or software that are central to the research but not yet described in published literature, software must be made available to editors and reviewers. We strongly encourage code deposition in a community repository (e.g. GitHub). See the Nature Research guidelines for submitting code & software for further information.

Data

Policy information about availability of data

All manuscripts must include a data availability statement. This statement should provide the following information, where applicable:
- Accession codes, unique identifiers, or web links for publicly available datasets
- A list of figures that have associated raw data
- A description of any restrictions on data availability

Researchers interested in obtaining an aggregated version of the data sufficient to reproduce the results reported in this paper should contact the corresponding author.

Field-specific reporting

Please select the one below that is the best fit for your research. If you are not sure, read the appropriate sections before making your selection.

☒ Life sciences ☐ Behavioural & social sciences ☐ Ecological, evolutionary & environmental sciences

For a reference copy of the document with all sections, see nature.com/documents/nr-reporting-summary-flat.pdf

Life sciences study design

All studies must disclose on these points even when the disclosure is negative.

Sample size	Based on the Pfizer vaccine's clinical trials we estimated at least 150 participants are required to ensure a sufficient amount of participants will present substantial reactions (such as fever). These subset of participants were likely to be identified as significantly different than the rest of the participants by the chest-patch sensor.
Data exclusions	Data collected from six participants were excluded; five left the trial early, and one was provided with a malfunctioning chest-patch sensor.
Replication	To validate the consistency of our findings, we split the data into two groups in a balanced fashion in terms of their age group and gender. One group contained 80%, and the other one contained the other 20%. The final analyses presented are based on the entire sample.
Randomization	A convenient sample. Findings were controlled by age and gender.
Blinding	Investigators were blinded during data collection and/or analysis.

Reporting for specific materials, systems and methods

We require information from authors about some types of materials, experimental systems and methods used in many studies. Here, indicate whether each material, system or method listed is relevant to your study. If you are not sure if a list item applies to your research, read the appropriate section before selecting a response.

Materials & experimental systems

n/a	Involved in the study	
☒	☐	Antibodies
☒	☐	Eukaryotic cell lines
☒	☐	Palaeontology and archaeology
☒	☐	Animals and other organisms
☐	☒	Human research participants
☐	☒	Clinical data
☒	☐	Dual use research of concern

Methods

n/a	Involved in the study	
☒	☐	ChIP-seq
☒	☐	Flow cytometry
☒	☐	MRI-based neuroimaging

Human research participants

Policy information about studies involving human research participants

Population characteristics	166 participants were recruited. Among them, 160 participants completed the trial, and their data were analyzed; Among these participants, 56.25% were females, 13.75% were obese (body-mass index of at least 30.0), 18.12% had high blood pressure, and 20.00% had at least one comorbidity. The median age was 40 years, and 10.63% of participants were older than 59 years of age. All participants were administered the BNT162b2 mRNA vaccine.
Recruitment	Eligible candidates to recruitment are individual aged > 18 years who were eligible to receive the vaccine.<br>To recruit participants and ensure they complete all the study's requirements, we hired a professional survey company. Participants were recruited through advertisements in social media, online banners, and word-of-mouth. The survey company was responsible for guaranteeing the participants meet the study's requirements, in particular, that they agree to wear the chest-patch sensor and fill in the daily questionnaires.
Ethics oversight	Tel-Aviv University Institutional Review Board

Note that full information on the approval of the study protocol must also be provided in the manuscript.

Clinical data

Policy information about clinical studies

All manuscripts should comply with the ICMJE guidelines for publication of clinical research and a completed CONSORT checklist must be included with all submissions.

Clinical trial registration	Not applicable.
Study protocol	Study protocol is available at the Supplementary Materials
Data collection	Participants were recruited during the mass vaccination campaign in Israel between 1 January, 2021 and 13 March, 2021. Chest-patch data were collected for four consecutive days, starting 24 hours before receiving the vaccine. Daily questionnaires data were collected for 15 days, starting 24 hours before receiving the vaccine.
Outcomes	Data regarding the 13 physiological parameters: heart rate, blood oxygen saturation, respiratory rate, systolic and diastolic blood pressure, pulse pressure, mean arterial pressure, heart rate variability, stroke volume, cardiac output, cardiac index, systemic vascular resistance, and body temperature. Additionally, data regarding self-reported questionnaire, stress level, sleep quality, physical activity, clinical symptoms).
